# Detection of co-infection and recombination cases with Omicron and local Delta variants of SARS-CoV-2 in Vietnam

**DOI:** 10.1038/s41598-024-64898-5

**Published:** 2024-06-20

**Authors:** Nguyen Thu Trang, Trinh Cong Dien, Nguyen Thi Tam, Phan Manh Cuong, Le Van Duyet, Nguyen Thi Hong Thuong, Van Dinh Trang, Pham Ngoc Thach, H. Rogier van Doorn, Thomas Kesteman, OUCRU COVID-19 research group, Chambers Mary, Choisy Marc, Dong Huu Khanh Trinh, Dong Thi Hoai Tam, Du Hong Duc, Dung Vu Tien Viet, Fisher Jaom, Flower Barney, Geskus Ronald, Hang Vu Thi Kim, Ho Quang Chanh, Ho Thi Bich Hai, Ho Van Hien, Hung Vu Bao, Huong Dang Thao, Huynh le Anh Huy, Huynh Ngan Ha, Huynh Trung Trieu, Huynh Xuan Yen, Kestelyn Evelyne, Kesteman Thomas, Lam Anh Nguyet, Lawson Katrina, Leigh Jones, Le Kim Thanh, Le Dinh Van Khoa, Le Thanh Hoang Nhat, Le Van Tan, Lewycka Sonia Odette, Lam Minh Yen, Le Nguyen Truc Nhu, Le Thi Hoang Lan, Nam Vinh Nguyen, Ngo Thi Hoa, Nguyen Bao Tran, Nguyen Duc Manh, Nguyen Hoang Yen, Nguyen Le Thao My, Nguyen Minh Nguyet, Nguyen To Anh, Nguyen Thanh Ha, Nguyen Than Ha Quyen, Nguyen Thanh Ngoc, Nguyen Thanh Thuy Nhien, Nguyen Thi Han Ny, Nguyen Thi Hong Thuong, Nguyen Thi Hong Yen, Nguyen Thi Huyen Trang, Nguyen Thi Kim Ngoc, Nguyen Thi Kim Tuyen, Nguyen Thi Ngoc Diep, Nguyen Thi Phuong Dung, Nguyen Thi Tam, Nguyen Thi Thu Hong, Nguyen Thu Trang, Nguyen Thuy Thuong Thuong, Nguyen Xuan Truong, Nhung Doan Phuong, Ninh Thi Thanh Van, Ong Phuc Thinh, Pham Ngoc Thanh, Phan Nguyen Quoc Khanh, Phung Ho Thi Kim, Phung Khanh Lam, Phung Le Kim Yen, Phung Tran Huy Nhat, Rahman Motiur, Thuong Nguyen Thi Huyen, Thwaites Guy, Thwaites Louise, Tran Bang Huyen, Tran Dong Thai Han, Tran Kim Van Anh, Tran Minh Hien, Tran Phuong Thao, Tran Tan Thanh, Tran Thi Bich Ngoc, Tran Thi Hang, Tran Tinh Hien, Trinh Son Tung, van Doorn H. Rogier, Van Nuil Jennifer, Vidaillac Celine Pascale, Vu Thi Ngoc Bich, Vu Thi Ty Hang, Yacoub Sophie

**Affiliations:** 1 Oxford University Clinical Research Unit, Hanoi, Vietnam; 2 Department of Infectious Diseases, Vietnam Military Medical University, Hanoi, Vietnam; 3 Departments of Infectious Disease, Military Hospital 103, Hanoi, Vietnam; 4 National Hospital for Tropical Diseases, Hanoi, Vietnam; 5 Centre for Tropical Diseases, Nuffield Department of Medicine, University of Oxford, Oxford, UK

**Keywords:** Microbiology, Virology

## Abstract

The first nationwide outbreak of COVID-19 in Vietnam started in late April 2021 and was caused almost exclusively by a single Delta lineage, AY.57. In early 2022, multiple Omicron variants co-circulated with Delta variants and quickly became dominant. The co-circulation of Delta and Omicron happened leading to possibility of co-infection and recombination events which can be revealed by viral genomic data. From January to October 2022, a total of 1028 viral RNA samples out of 4852 positive samples (Ct < 30) were sequenced by the long pooled amplicons method on Illumina platforms. All sequencing data was analysed by the workflow for SARS-CoV-2 on CLC genomics workbench and Illumina Dragen Covid application. Among those sequenced samples, we detected a case of Delta AY.57/Omicron BA.1 co-infection and two cases of infection with Delta AY.57/Omicron BA.2 recombinants which were nearly identical and had different epidemiological characteristics. Since the AY.57 lineage circulated almost exclusively in Vietnam, these results strongly suggest domestic events of co-infection and recombination. These findings highlight the strengths of genomic surveillance in monitoring the circulating variants in the community enabling rapid identification of viral changes that may affect viral properties and evolutionary events.

## Introduction

All viruses, including SARS-CoV-2, change over time by mutations and some can lead to new variants. During late 2020, the emergence of variants that posed an increased risk to global public health prompted the characterization of specific Variants of Concern (VOCs), in order to prioritize global monitoring, research and ultimately to inform the ongoing response to the COVID-19 pandemic. By 31 May 2021, WHO labeled five SARS-CoV-2 variants as VOCs including Alpha (lineage B.1.1.7), Beta (lineage B.1.351), Gamma (lineage P.1), Delta (lineage B.1.617.2), and Omicron (lineage B.1.1.529), and updated its tracking system and working definitions of VOCs, VOIs (Variant of Interest) and VUM (Variant Under Monitoring) on 15 March 2023^1,2^.

**Figure 1:**
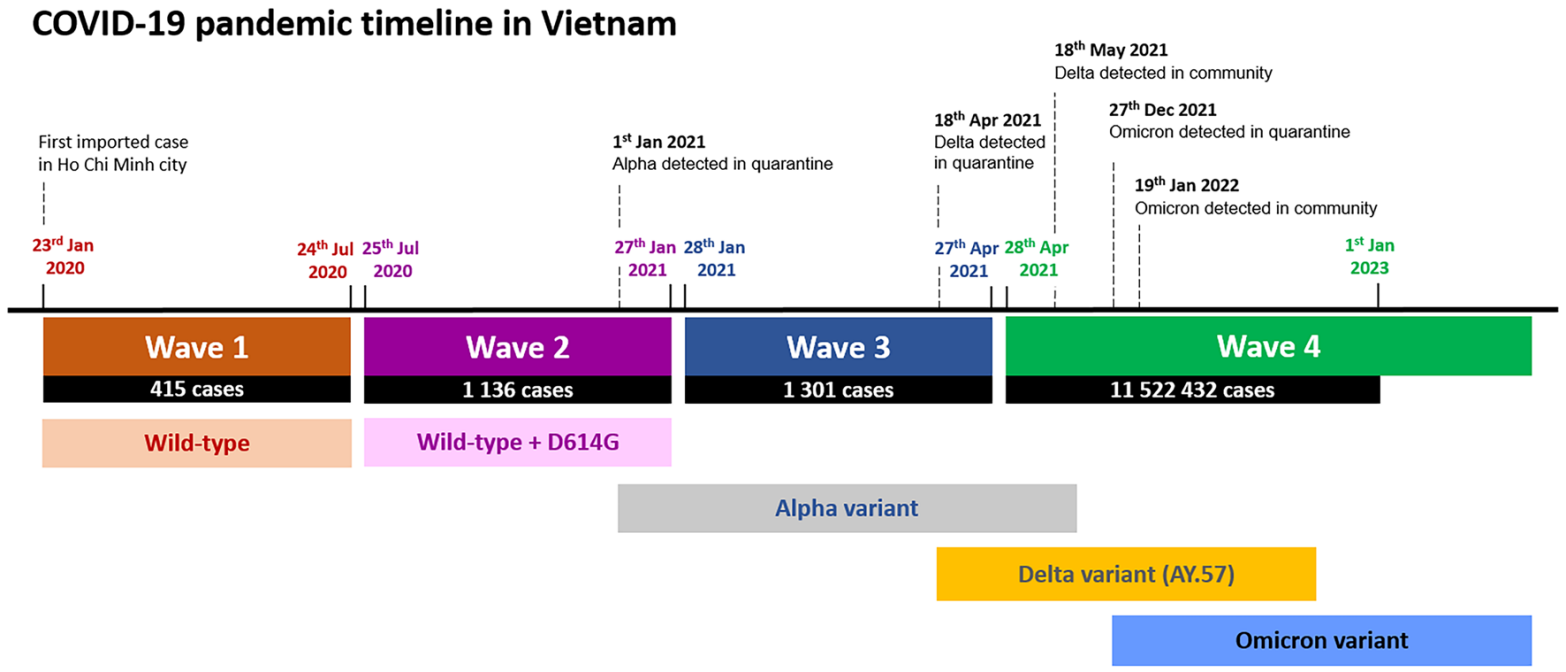
COVID-19 pandemic timeline in Vietnam. The figure shows main events of fourth waves of COVID-19 pandemic in Vietnam.

Vietnam had experienced three locally contained COVID-19 clusters between January 2020 and April 2021, with over 2800 cases and 35 deaths during that time^3,4^. The fourth cluster became the first real wave in late April 2021, caused by a fast spreading Delta variant, leading to several thousands of cases confirmed daily from July onwards (Fig. 1). Lineage AY.57 accounted for 99% of Delta sublineages circulating in Vietnam in 2021^4^. During the Delta outbreak, Vietnam implemented a strict national lockdown with very limited domestic travel, and further tightened border controls. At that time, AY.57 was almost the only sub-lineage circulating in Vietnam and once AY.57 was established, sporadic introductions of other Delta lineages remained trivial, until the arrival of the more transmissible Omicron variant in early 2022^4^. The Delta AY.57 lineage was almost specific to Vietnam as it was only sporadically detected outside of Vietnam (92.9% of AY.57 sequences on GISAID in 2021 were from Vietnam).

On 24 November 2021, the Omicron variant was first detected in South Africa with many novel mutations, especially in the spike protein^5,6^. On 27 December 2021, the Vietnamese Ministry of Health confirmed the first Omicron imported case, which was followed by numerous other importation events^7^. At that time, the Delta AY.57 variant was still dominant and had caused nearly 1.8 million cases and over 32.000 deaths across the country^8^. One month after the first imported case, several Omicron sub-lineages (mainly BA) widely circulated in the community and, in Northern Vietnam, Omicron had overtaken Delta as the dominant variant by the end of February 2022^9,10^. According to GISAID database, Delta AY.57 was not reported in Vietnam since July 2022.

A period of co-circulation of two distinct VOCs allowed co-infection and recombination to happen but these events have not been reported in Vietnam. From 2020 to 2023, we carried out the SARS-CoV-2 genomic surveillance by whole genome sequencing at the tertiary referral hospital for COVID-19 patients in Northern Vietnam. Here, we describe cases of Delta – Omicron coinfection and recombination detected in 2022, and the genetic characteristics and clinical features of these variants.

## Results

### Co-circulation of *Delta* and Omicron variants

**Figure 2:**
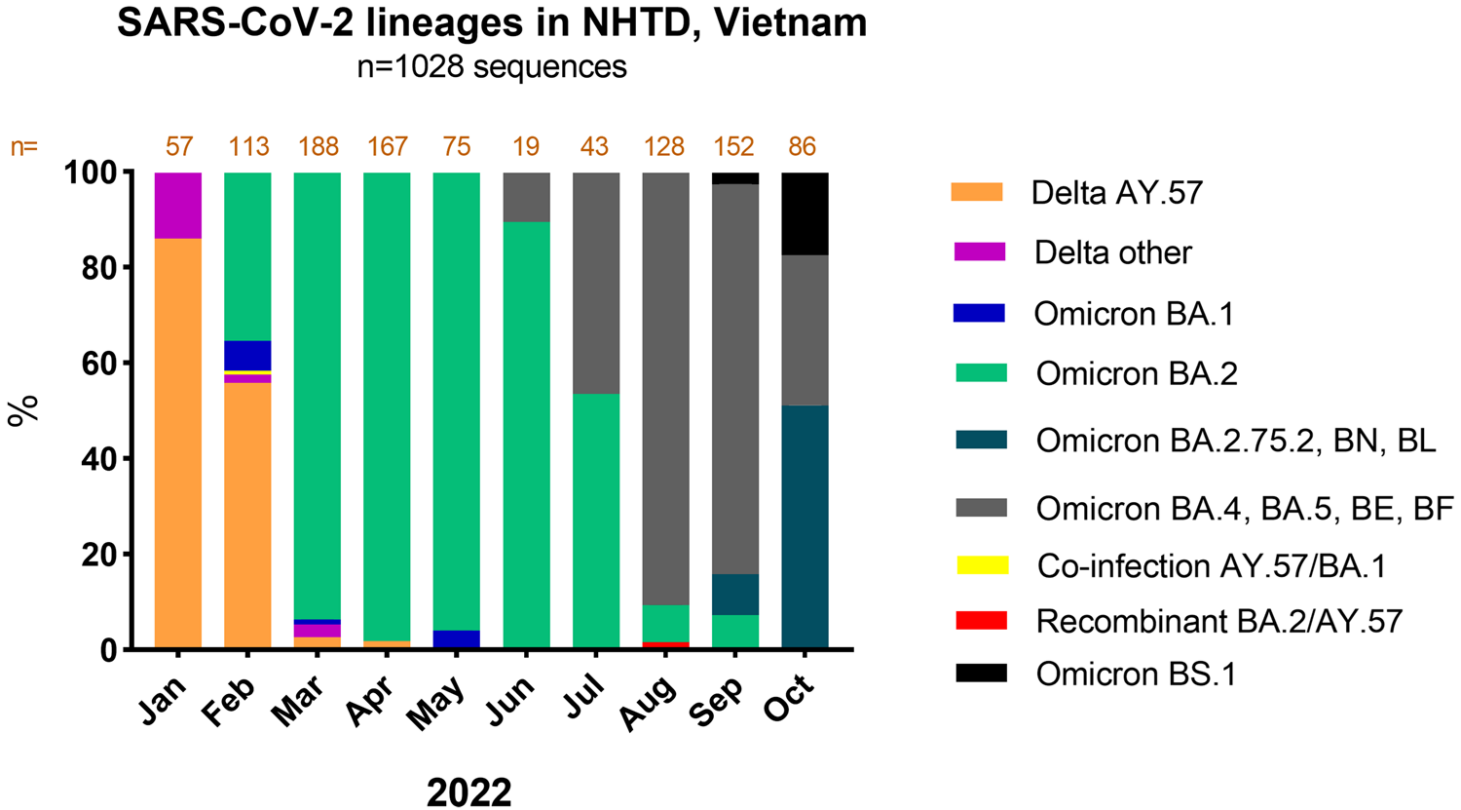
Distribution of circulating variants between January and October 2022 from the patients at NHTD, Vietnam.

From January to October 2022, a total of 1,028 SARS-CoV-2 samples were sequenced. Patients came from 39 different provinces, 88.9% of them came from the North of Vietnam, specifically 28.5% from Hanoi city, 1.7% from Thai Nguyen province, 2.0% from Hai Phong city, and 6.5% from Hai Duong province. The samples included the different lineages of Delta and Omicron as described in Fig. 2. All sequences in January 2022 belonged to Delta sublineages, mainly AY.57 accounting for 85%. Omicron sequences accounted for 42% in February, increased to 95% in March, and 98% in April. We detected one case of co-infection by Delta AY.57/Omicron BA.1 in February when these two variants co-circulated. From May onwards, only Omicron sublineage was found, with the exception of two infections by Delta AY.57/Omicron BA.2 recombinant viruses in August, six months after the detection of the co-infection and three months after the Delta variant was totally overtaken by the Omicron variant.

### Co-infection of *Delta* and Omicron variants

**Figure 3:**
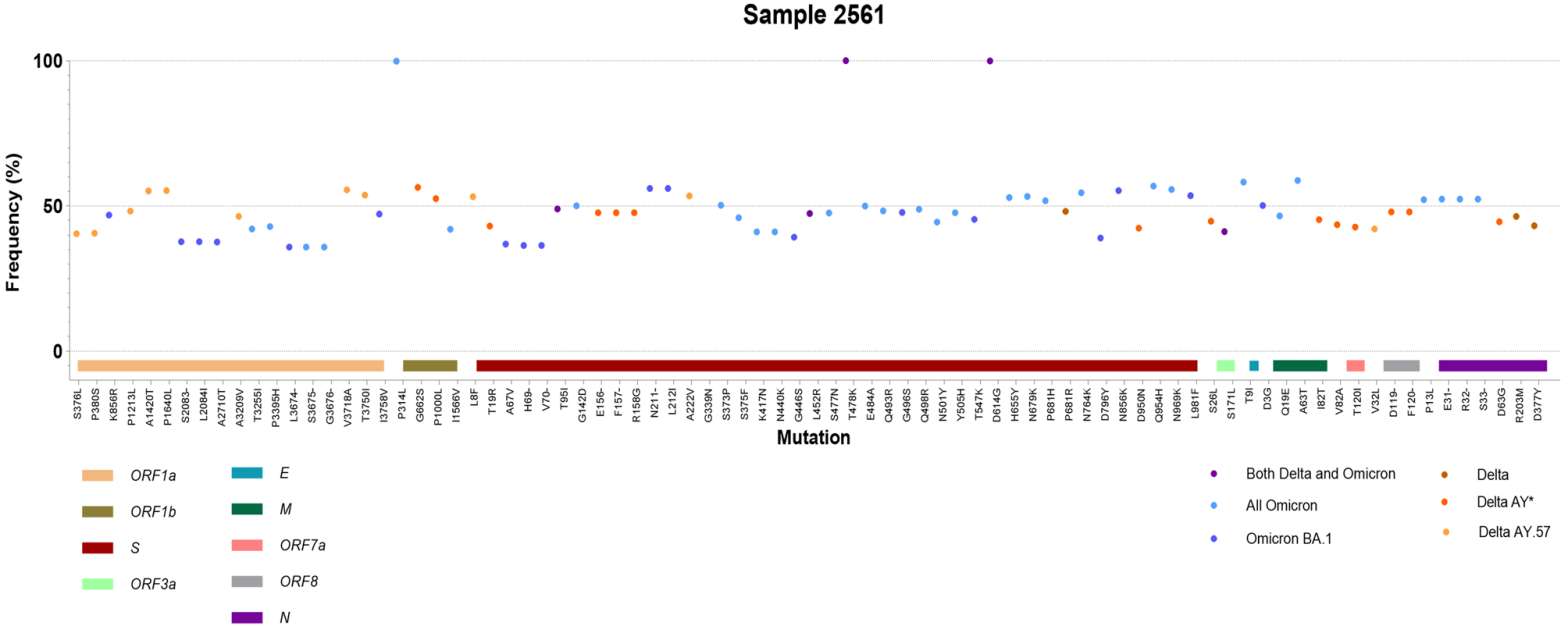
Co-infection with Delta and Omicron variants. The graph shows the frequency and relative location of each specific mutation of each variant type along the viral genome (GISAID accession No. EPI_ISL_ 11632985). The coloured dots represent the specificity of the mutations: light orange for mutations of Delta AY.57; orange for mutations of Delta AY*; light brown for common mutations of all Delta; navy blue for mutations of Omicron BA.1; light blue for common mutaions of all Omicron; purple for mutation shared by both Delta and Omicron. The vertical axis is the frequency of the mutation. The x axis is divided into functional regions of SARS-CoV-2 and highlighted by corresponding colours.

Here, we detected one case of coinfection of Delta/Omicron from a patient admitted at the NHTD on 17 February 2022. Sequencing data of this patient, labelled as #2561, identified both Delta- and Omicron-specific mutations. The consensus sequence was constructed with high coverage (2403x ± 1219). The mutations specific for Delta and Omicron and most other mutations detected in this sample were present at frequency around 50% (range: 35.9%-58.8%, Fig. 3 and Table S1). In addition, mutations of each variant type were mixed along entirety of the viral genome, and were not located together on a certain region of the genome (Fig. 3). Apart from the common marker mutations for Delta and Omicron, we also observed the presence of specific mutations for the Delta AY.57 and the Omicron BA.1 (Additional information, Table S1). Therefore, this case was most likely a co-infection of an AY.57 and a BA.1 lineage.

### *Delta*/Omicron recombinant infection

**Figure 4:**
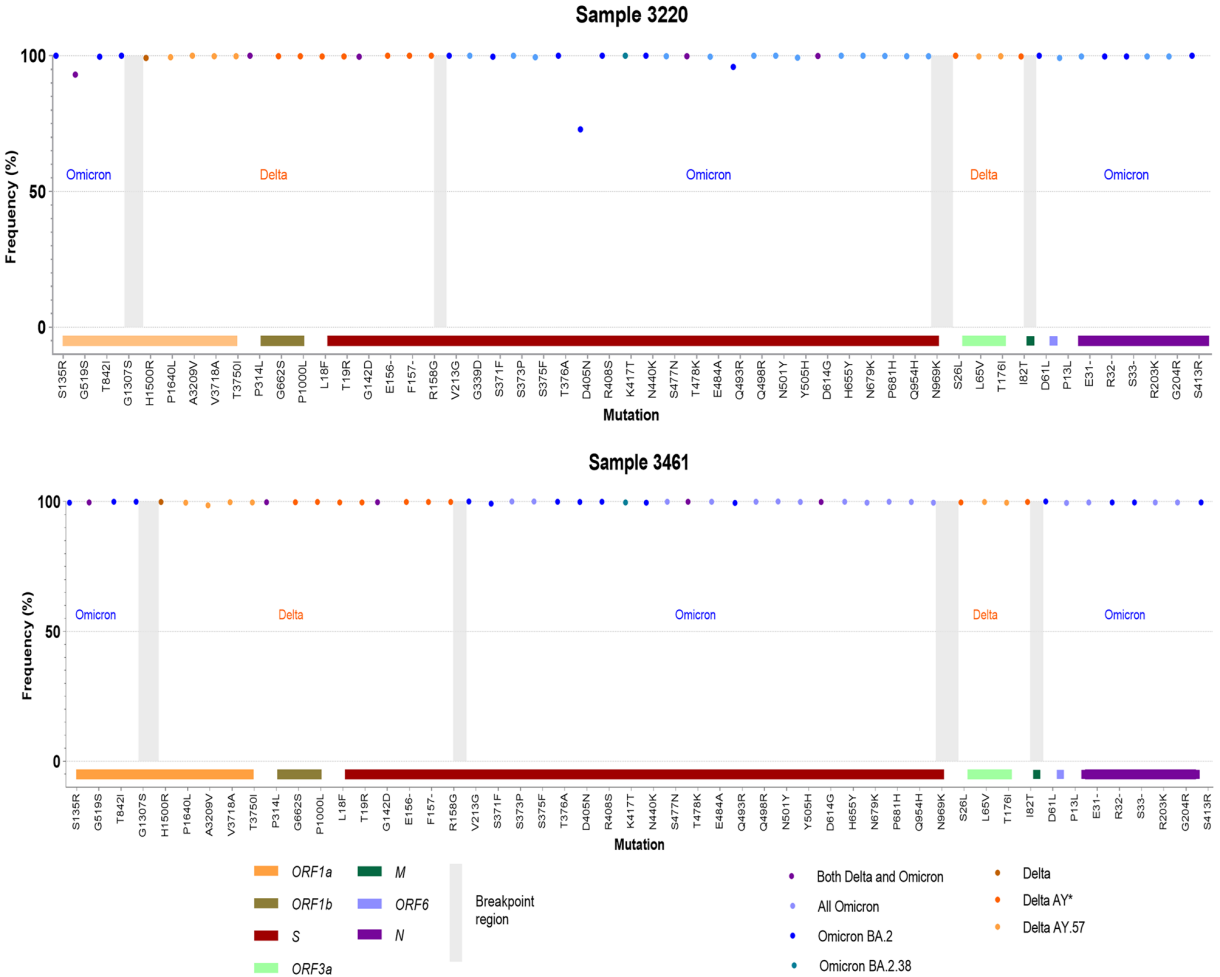
Recombination of Delta and Omicron variants. The graph shows the frequency and relative location of each specific mutation of each variant type along the viral genome (GISAID accession No.: EPI_ISL_14934965, EPI_ISL_15579330 for sample 3220 and 3461 respectively). The coloured dots represent the specificity of the mutations: light orange for mutations of Delta AY.57; orange for mutations of Delta AY*; light brown for common mutations of all Delta; navy blue for mutations of Omicron BA.1; light blue for common mutaions of all Omicron; purple for mutation shared by both Delta and Omicron. The vertical axis is the frequency of the mutation. The x axis is divided into functional regions of SARS-CoV-2 and highlighted by corresponding colorus.

In August 2022, different sequences from two patients harbouring both Delta AY.57 and Omicron BA.2 markers were detected. The first case was sampled on 15 August 2022 (sample 3220) and the second case two weeks later, on 29 August 2022 (sample 3461). Sequencing results showed similar viral sequences, with just one difference at nucleotide position 17,459 C > T, leading to an amino acid change (nsp13:P408L). The consensus sequence of both samples was reliable with high coverage (1015x ± 500 for the first sample and 2327x ± 1111 for the second sample). The frequency of VOC-specific mutations ranged from 93 to 100%, except a mutation D405N on the *S* region of the first sample (73%) (Fig. 4). The mutations specific for either Delta or Omicron variants were presented in five separate regions of the genome, suggesting a recombination event with (at least) four breakpoints (Fig. 4). In addition, we found that each pair of Delta and Omicron mutations around breakpoints were both present on single reads covering both regions, thus excluding the possibility of a technical artifact. For example, the mutation G1307S (Omicron) and H1500R (Delta) in the first breakpoint region are present on all of the seven raw reads covering both regions (Figure S2). The mutation types and their frequency from these two samples are listed in the additional information, Table S2.

We constructed artificial sequences containing either only Delta fragments or only Omicron fragments by replacing all bases of Omicron regions or Delta on genome respectively with “N” for both samples. These sequences were subsequently uploaded on Nextclade v2.9.1^11^ to assign clade and identify position of these artificial sequences on the global Nextclade phylogenetic tree. As a result, the sequences with only Delta fragments were closest to sublineage Delta AY.57 belonging to the clade 21A and the sequences with only Omicron fragments were closest to the Omicron BA.2.40.1 belonging to the clade 21L (Additional information, Figure S1). This analysis confirmed that the recombinant viruses in our study resulted from hybridization of sublineages Delta AY.57 and of Omicron BA.2.

### Clinical and epidemiological features of co-infection and recombination cases

The patient co-infected with Delta AY.57/Omicron BA.1 was a 78-year-old woman from Thai Nguyen province with no history of recent travel outside the province, and therefore her infection likely happened locally. Although the patient was elderly and had hypertension, the symptoms before admission were mild including low-grade of fever, cough, sore throat, fatigue, chest pain, and mild dyspnea (Table 1). The patient’s condition remained stable during hospitalization without oxygen support. Most of laboratory tests were within normal ranges including both CRP (C-reactive protein, normal < 10 mg/L) and LDH (Lactate dehydrogenase, range from 230 to 460 U/L). Only the D-Dimer was increased slightly but no thrombotic events were noted. The patient had received 3 doses of the COVID-19 vaccine prior to infection.

**Table 1:** Main characteristics of the patients with Delta-Omicron co-infection and recombination.

Sample	Age group (years)	Gender	Province/city	Collection date	Vaccine dose	Comorbidities	Clinical form*	Severity	Lineage
2561	70–80	Female	Thai Nguyen	17/02/2022	3 doses	Hypertension	ILI	Mild/ Moderate	Co-infection AY.57/ BA.1
3220	40–50	Male	Hai Phong	16/08/2022	Unvaccinated	Malignancy	ILI	Mild/ Moderate	Recombinant AY.57/BA.2
3461	70–80	Female	Hai Duong	29/08/2022	3 doses	Chronic anemia	SARI	Severe/Dead	Recombinant AY.57/BA.2

*ILI: Influenza-Like Illness; SARI: Severe Acute Respiratory Infections.

The first patient with the Delta AY.57/Omicron BA.2 recombinant was a 47-year-old man from Hai Phong city with no SARS-CoV-2 infection documented previously and no prior vaccination (Table 1). The patient had Peripheral T-cell lymphoma and had received treatment with Induced Pluripotent Stem Cells since 2020. His symptoms were mild with fever and cough only. Some hematological and biochemical tests were slightly out of the normal range, and the CRP was also elevated (47 mg/L). Chest CT scan showed ground-glass opacity in both lower lung lobes. The LDH and D-Dimer were in the normal range and there were no other risk factors. His situation improved quickly without oxygen support, and he was discharged after over one week of admission.

The second patient infected with the recombinant virus was a 76-year-old woman from Hai Duong province who had not traveled outside her accommodation, so the infection source was probable inside the province. Hai Duong province borders Hai Phong city where the first patient lived. This suggests community circulation of this Delta AY.57/Omicron BA.2 recombinant in this area. This patient had received three doses of COVID-19 vaccine, and had several severe symptoms before provincial hospital admission including intermittent fever, cough, diarrhea, poor eating, jaundice and dyspnea (Table 1). Although she received treatment during hospitalization including oxygen support and blood transfusion (for chronic anemia), her dyspnea worsened and endotracheal intubation was required. Therefore, she was transferred to NHTD for further tests and treatment. Her condition did not improve and she developed symptoms of organ failure and hypercoagulability and was transferred to intensive care unit (ICU ward). After nearly one week of treatment, she died of ARDS (Acute Respiratory Distress Syndrome).

## Discussion

In this study, we identified one case of co-infection of Delta AY.57/Omicron BA.1 and two cases of infection with Delta AY.57/Omicron BA.2 recombination. This is also the first report about these events in Vietnam and one contributing variant was the local Delta variant AY.57 which suggested that the co-infection and recombination were domestic. To identify the co-infected and recombined genomes without experimental errors, we used a double pipeline to analyze the sequencing data, which each application would support the other, especially mutation detection. We, therefore, can confirm the quality of all sequences and their mutations by checking all QC indicators, the coverage and the frequency of all mutations.

We hypothesized two scenarios resulting in the co-infection. The first scenario is that the patient was infected by Delta AY.57 and Omicron BA.1 at the same time. The second possibility involves sequential infections from different sources. However, the limited epidemiological information and biological samples received could not help us discerning these two hypotheses. The time we detected the co-infection case (17 January 2022) is also in the co-circulation period of Delta and Omicron (mainly from January to April 2022 which was consistent with data from Vietnam submitted to GISAID in 2022). The occurrence of simultaneous infection by two distinct lineages creates an opportunity for viral genetic recombinations and emergence of new lineages with different characteristics^12^. Therefore, the recombinant Delta AY.57/Omicron BA.2 probably emerged between January and April, then circulated silently and were detected only later on. Given the small number of samples sequenced per all COVID-19 samples in Vietnam, the surveillance network probably missed the possible recombinant viruses having emerged in the country before August. Besides, our method may have missed co-infections with strong imbalance in co-infecting variants as it would have generated a consensus sequence representing the dominant variant only.

Notably, the national vaccination proportion was high when Omicron variant spread in the community. As of 6 February 2022, a total of 183,196,831 doses have been administered: 79,148,668 people from 12 years of age and above completed the first dose (~ 80.6% of total population) and 74,356,060 of them completed the second dose (~ 75.7% of total population)^13^. Therefore, the co-circulation of Delta and Omicron variants and the emergence of recombinant viruses happened at a time of high vaccination coverage, and yet the recombinant virus circulated marginally. This suggested that high vaccination coverage does not increase the probability of selection of recombinants.

The two recombinant viruses described here were from different patients, collected at different time points (13 days apart) and locations (Hai Duong province and Hai Phong city), and we have no evidence connecting these cases in terms of epidemiology. However, their nearly identical viral genomes, as revealed by whole genome sequencing data, suggests that they have a shared source. Our clinical data indicated that the two patients lived in two nearby provinces, and were infected at home. Community circulation of this Delta AY.57/Omicron BA.2 recombinant virus in this area is therefore likely to appear in this area, but did not spill over to other provinces. To our knowledge, these cases are not only the first description of a recombination between the Delta AY.57 and the Omicron BA.2 variants, but also a rare description of a recombinant with four breakpoints. Recombinants usually have one or two breakpoints; recombinants with more breakpoints have been described, such as XAY in South Africa that has six breakpoints and several unique mutations not found in the parental lineages^14,15^. It was hypothesized that XAY resulted from a chronic Delta infection with BA.2 and iterative recombination before being seeded back to the general population. The relative paucity of unique mutations in the cases presented here might point to a different mechanism, possibly a shorter co-infection time in the index patient^15,16^.

The evolution of three recombinant lineages of SARS-CoV-2 is mainly XD (Delta and BA.1), XE (BA.1 and BA.2), and XF (Delta and BA.1), which shows probably a high degree of transmissibility. The XE recombinant was found on 19 January 2022 and widely spread throughout London before it was outcompeted by BA.5. The BA.2 shows a 75% growth rate in comparison with BA.1, XE was noted to have a 9.8% higher growth rate as compared to the BA.2 sub-lineage of the Omicron^17^. This suggests that the recombinant viral strains may potentially be reflecting a short transmission chain after an imported event. The recombination of Delta and BA.2 was not commonly detected except for a recent notable lineage XAY, which was first discovered in South Africa in May 2022 alongside a smaller sister recombinant, XBA^15^ and our case reports. However, not all viral recombination could show possibly high rate of transmission. No other recombinants were detected in our surveillance. The recombinants were found in August, when the dominance of BA.2 was warning and other variants became dominant (BA.4, BA.5, BE, BF) (Fig. 2). We found no evidence that the detected viral recombinants have any important selective advantages to outcompete other circulating variants.

In late 2022, XBB emerged through the recombination of two co-circulating BA.2 lineages, BJ.1 and BM.1.1.1 (a progeny of BA.2.75) with one breakpoint located in the receptor-binding domain of spike which conferred immune evasion and increased fusogenicity^18^. After that, in December 2022, the uncommon presentation of XBL variant (XBB.1 and BA.2.75) as the first recombinant of a recombinant opened the second generation of SARS-CoV-2 recombination^19,20^. Well understanding of SARS-CoV-2 recombinant lineages provides us with vaccine preparedness, especially when further-generation recombination could bring more characteristics for immune escape and lead to increased disease severity.

## Methods

### Sample collection and SARS-CoV-2 diagnostics

Our study was conducted at the National Hospital for Tropical Diseases in Hanoi, Vietnam. A tertiary referral centre for isolation and treatment of COVID-19 patients. Nasopharyngeal swabs of all suspected COVID-19 patients were collected from January to October 2022 in viral transport medium and tested by real-time RT-PCR within 24 h of collection. Viral RNA was extracted automatically using MagNA Pure 96 (Roche, Basel, Switzerland), SARS-CoV-2 was confirmed by the presence of the *E* gene (112 bp) and RNA-dependent RNA polymerase (*RdRp*) gene (99 bp) using SuperScript III Platinum One-Step qRT-PCR kit (Invitrogen, Carlsbad, CA USA) and E-Sarbeco, RdRp primers and probes (Tib Molbiol, Berlin, Germany ) as described previously^21^. Positive samples with cycle threshold (Ct) values under 30 for both genes were stored at -80˚C for whole genome sequencing.

### Whole genome sequencing

The selection of samples for whole genome sequencing based on WHO recommendations to reflect the representativeness of sampled patients (i.e., geographical origin, age and sex, disease severity, timepoint during the outbreak)^22^. We sequenced 1028 viral RNA samples out of 4852 positive samples with Ct < 30 between January and October 2022. The selected samples then were sequenced following the long pooled amplicons protocol, developed by the University of Sydney^23^ on Illumina Miseq platform using Nextera XT Library preparation kit, Nextera XT index kit v2 and the 300 cycle V2 kit (Illumina, USA)^24^. For samples suspected being co-infection and recombination, their RNA was re-extracted using QIAamp viral mini kit (Qiagen) and whole genome sequencing was repeated on Illumina platform. Result of replication was to confirm no contamination during sample processing and sequencing.

### Data analysis

Raw data generated from the Miseq sequencer (.fastq files) was analysed by two pipelines: work-flow for SARS-CoV-2 on CLC genomics workbench (Qiagen)^25^ and Illumina Dragen Covid application available on the BaseSpace cloud (https://basespace.illumina.com)^26^.

The Illumina® DRAGEN COVID Lineage App (version 3.5.13) on Illumina BaseSpace performs Kmer-based detection of SARS-CoV-2 by aligning reads to a reference genome (NC_045512) to generate a consensus genome sequence, then call variants and identify lineage/clade using Pangolin and NextClade^27^. The consensus sequences generated from DRAGEN were uploaded to GISAID.

We also used the ‘Identify QIAseq SARS-CoV-2 Low Frequency and Shared Variants (Illumina) v1.53’ workflow performed on CLC genomics workbench version 22.0.2 to identify SARS-CoV-2 mutations by firstly trimming adapters and primers and filtering short reads (≤ 50 bp), then mapping trimmed reads to the SARS-CoV-2 reference genome (NC_045512) to produce a viral consensus sequence, finally calling high quality variant. The advantage of this variant calling method was to show frequency and coverage of each modification, therefore we could confirm a true mutation with threshold of 70% of variant allele frequency and at least 30 × coverage. In a sample identified as a case of coinfection, mutations at signature positions defining two population lineages are expected to co-exist, with variable frequencies, depending on the relative share of each lineage present in the sample. Frequencies occurring at 40–60% and multiple alleles accounting for > 50% of all mutations detected in a sample are strongly suggestive of a coinfection event. The co-infection by genetically distinct viruses in the same cells within an individual can lead to viral recombination due to a high chance during genome replication that the polymerase will switch from one genome sequence template to the heterologous genome^15^. The recombinant viral genome is expected to carry specific mutations of each SARS-CoV-2 variant at frequency around 100%, as opposed to co-infections^28,29^.

We used Qualimap software^30^ to evaluate sequencing depth across the reference genome and calculate mean coverage values.

We used the GraphPad Prism software version 8.3.0 to statistically analyse of mutations and build figures^31^.

### Ethics approval and consent to participate

This study was approved by the Ethic Committee of National Hospital for Tropical Disease (NHTD), Vietnam (Decision No. 17-2022/HDDD-NDTU), and the need for participant consent was waived by the Ethics Committee of NHTD. All methods were carried out in accordance with Vietnamese guidelines and regulations.

### Consent for publication

All authors read and approved the final manuscript.

### Supplementary Information

Supplementary Information 1.

Supplementary Information 2.

Supplementary Information 3.

## Data Availability

All consensus sequences of 1028 samples were submited to GISAID (accssion number EPI_ISL_10070370–EPI_ISL_10070426; EPI_ISL_10566247–EPI_ISL_10566301; EPI_ISL_11221195–EPI_ISL_11221249; EPI_ISL_11632985; EPI_ISL_12252511–EPI_ISL_12252574; EPI_ISL_12305030–EPI_ISL_12305093; EPI_ISL_12305800; EPI_ISL_12309119–EPI_ISL_12309178; EPI_ISL_13294630–EPI_ISL_13294675; EPI_ISL_13294600–EPI_ISL_13294627; EPI_ISL_13294550–EPI_ISL_13294598; EPI_ISL_13294494–EPI_ISL_13294548; EPI_ISL_13298593; EPI_ISL_13358757–EPI_ISL_13358813; EPI_ISL_14691857–EPI_ISL_14701320; EPI_ISL_14934946–EPI_ISL_14934965; EPI_ISL_15479807–EPI_ISL_15504801; EPI_ISL_15579277–EPI_ISL_15579329; EPI_ISL_15579330–EPI_ISL_15579338; EPI_ISL_15579547–EPI_ISL_15579553; EPI_ISL_15887414–EPI_ISL_15887458; EPI_ISL_15887460–EPI_ISL_15887464; EPI_ISL_15887604–EPI_ISL_15887639; EPI_ISL_15887641–EPI_ISL_15887669; EPI_ISL_15894745–EPI_ISL_15894752; EPI_ISL_15894943–EPI_ISL_15894956; EPI_ISL_16865540; EPI_ISL_16449433–EPI_ISL_16449502; EPI_ISL_16472477–EPI_ISL_16472481).
