# Supplementary material for: Detection of co-infection and recombination cases with Omicron and local Delta variants of SARS-CoV-2 in Vietnam
Source: Sci Rep. 2024 Jun 20;14:14225. doi: 10.1038/s41598-024-64898-5 (PMC11190198; doi:10.1038/s41598-024-64898-5)
Supplement: Supplementary file 1 — Supplementary Information 1. [file 41598_2024_64898_MOESM1_ESM.docx]

**Detection of co-infection and recombination cases with Omicron and local Delta variants of SARS-CoV-2 in Vietnam**

Nguyen Thu Trang^1,#,*^, Trinh Cong Dien^2,3,#^, Nguyen Thi Tam^1^, Phan Manh Cuong^4^, Le Van Duyet^4^, Nguyen Thi Hong Thuong^1^, Van Dinh Trang^4^, Pham Ngoc Thach^4^, H. Rogier van Doorn^1,5^, Thomas Kesteman^1,5^, for OUCRU COVID-19 research group

^1^Oxford University Clinical Research Unit, Hanoi, Vietnam

^2^Department of Infectious diseases, Vietnam Military Medical University, Hanoi, Vietnam

^3^Departments of Infectious Disease, Military Hospital 103, Hanoi, Viet Nam

^4^National Hospital for Tropical Diseases, Hanoi, Vietnam

^5^Centre for Tropical Diseases, Nuffield Department of medicine, University of Oxford, Oxford, UK

^#^ Contributed equally

*Corresponding authors: Nguyen Thu Trang ([trangnt@oucru.org](mailto:trangnt@oucru.org))

Members of the working group are presented in the Acknowledgements

**Supplementary Information**

**Supplementary tables**

**Table S1. List of detected mutations in co-infection case**

| **Sample** | **Region** | **Gene** | **Acid amin change** | **Type*** | **Reference** | **Allele** | **Coverage** | **Frequency (%)** | **Variant** |
| --- | --- | --- | --- | --- | --- | --- | --- | --- | --- |
| 2561 | 1392 | ORF1a | S376L | SNV | C | T | 1420 | 40.49 | Delta AY.57 |
| 2561 | 1403 | ORF1a | P380S | SNV | C | T | 1444 | 40.65 | Delta AY.57 |
| 2561 | 2832 | ORF1a | K856R | SNV | A | G | 2196 | 46.86 | Omicron BA.1 |
| 2561 | 3903 | ORF1a | P1213L | SNV | C | T | 1127 | 48.27 | Delta AY.57 |
| 2561 | 4523 | ORF1a | A1420T | SNV | G | A | 837 | 55.20 | Delta AY.57 |
| 2561 | 5184 | ORF1a | P1640L | SNV | C | T | 987 | 55.32 | Delta AY.57 |
| 2561 | 6513..6515 | ORF1a | S2083- | Deletion | GTT | - | 697 | 37.73 | Omicron BA.1 |
| 2561 |  | ORF1a | L2084I |  |  |  |  |  | Omicron BA.1 |
| 2561 | 8393 | ORF1a | A2710T | SNV | G | A | 1492 | 37.60 | Omicron BA.1 |
| 2561 | 9891 | ORF1a | A3209V | SNV | C | T | 446 | 46.41 | Delta AY.57 |
| 2561 | 10029 | ORF1a | T3255I | SNV | C | T | 1790 | 42.07 | All Omicron |
| 2561 | 10449 | ORF1a | P3395H | SNV | C | A | 3157 | 42.95 | All Omicron |
| 2561 | 11283..11291 | ORF1a | L3674- | Deletion | GTTTGTCTG | - | 2106 | 35.85 | Omicron BA.1 |
| 2561 |  | ORF1a | S3675- |  |  |  |  |  | All Omicron |
| 2561 |  | ORF1a | G3676- |  |  |  |  |  | All Omicron |
| 2561 | 11418 | ORF1a | V3718A | SNV | T | C | 1818 | 55.56 | Delta AY.57 |
| 2561 | 11514 | ORF1a | T3750I | SNV | C | T | 1706 | 53.75 | Delta AY.57 |
| 2561 | 11537 | ORF1a | I3758V | SNV | A | G | 1684 | 47.27 | Omicron BA.1 |
| 2561 | 14408 | ORF1b | P314L | SNV | C | T | 1483 | 99.87 | All Omicron |
| 2561 | 15451 | ORF1b | G662S | SNV | G | A | 1073 | 56.38 | Delta AY* |
| 2561 | 16466 | ORF1b | P1000L | SNV | C | T | 590 | 52.54 | Delta AY* |
| 2561 | 18163 | ORF1b | I1566V | SNV | A | G | 2108 | 42.03 | All Omicron |
| 2561 | 21586 | S | L8F | SNV | G | T | 511 | 53.23 | Delta AY.57 |
| 2561 | 21618 | S | T19R | SNV | C | G | 1170 | 43.08 | Delta AY* |
| 2561 | 21762 | S | A67V | SNV | C | T | 1478 | 36.87 | Omicron BA.1 |
| 2561 | 21765..21770 | S | H69- | Deletion | TACATG | - | 1467 | 36.40 | Omicron BA.1 |
| 2561 |  | S | V70- |  |  |  |  |  | Omicron BA.1 |
| 2561 | 21846 | S | T95I | SNV | C | T | 1070 | 48.97 | Both Delta and Omicron |
| 2561 | 21987 | S | G142D | SNV | G | A | 421 | 50.12 | All Omicron |
| 2561 | 22029..22034 | S | E156- | Deletion | AGTTCA | - | 499 | 47.70 | Delta AY* |
| 2561 |  | S | F157- |  |  |  |  |  | Delta AY* |
| 2561 |  | S | R158G |  |  |  |  |  | Delta AY* |
| 2561 | 22194..22196 | S | N211- | Deletion | ATT | - | 700 | 56.00 | Omicron BA.1 |
| 2561 |  | S | L212I |  |  |  |  |  | Omicron BA.1 |
| 2561 | 22227 | S | A222V | SNV | C | T | 658 | 53.50 | Delta AY.57 |
| 2561 | 22578 | S | G339N | SNV | G | A | 642 | 47.98 | Omicron |
| 2561 | 22673..22674 | S | S371P | MNV | TC | CT | 623 | 44.78 | NA |
| 2561 | 22679 | S | S373P | SNV | T | C | 551 | 50.27 | All Omicron |
| 2561 | 22686 | S | S375F | SNV | C | T | 594 | 45.96 | All Omicron |
| 2561 | 22813 | S | K417N | SNV | G | T | 479 | 41.13 | All Omicron |
| 2561 | 22882 | S | N440K | SNV | T | G | 353 | 41.08 | All Omicron |
| 2561 | 22898 | S | G446S | SNV | G | A | 374 | 39.30 | Omicron BA.1 |
| 2561 | 22917 | S | L452R | SNV | T | G | 620 | 47.42 | Both Delta and Omicron |
| 2561 | 22992 | S | S477N | SNV | G | A | 874 | 47.60 | All Omicron |
| 2561 | 22995 | S | T478K | SNV | C | A | 874 | 100.00 | Both Delta and Omicron |
| 2561 | 23013 | S | E484A | SNV | A | C | 838 | 50.00 | All Omicron |
| 2561 | 23040 | S | Q493R | SNV | A | G | 753 | 48.34 | All Omicron |
| 2561 | 23048 | S | G496S | SNV | G | A | 778 | 47.81 | Omicron BA.1 |
| 2561 | 23055 | S | Q498R | SNV | A | G | 765 | 48.89 | All Omicron |
| 2561 | 23063 | S | N501Y | SNV | A | T | 1019 | 44.46 | All Omicron |
| 2561 | 23075 | S | Y505H | SNV | T | C | 963 | 47.66 | All Omicron |
| 2561 | 23202 | S | T547K | SNV | C | A | 2306 | 45.40 | Omicron BA.1 |
| 2561 | 23403 | S | D614G | SNV | A | G | 4063 | 99.98 | Both Delta and Omicron |
| 2561 | 23525 | S | H655Y | SNV | C | T | 4148 | 52.97 | All Omicron |
| 2561 | 23599 | S | N679K | SNV | T | G | 3740 | 53.34 | All Omicron |
| 2561 | 23604 | S | P681H | SNV | C | A | 3441 | 51.85 | All Omicron |
| 2561 | 23604 | S | P681R | SNV | C | G | 3441 | 48.13 | Delta |
| 2561 | 23854 | S | N764K | SNV | C | A | 1531 | 54.60 | All Omicron |
| 2561 | 23948 | S | N856K | SNV | G | T | 894 | 39.04 | Omicron BA.1 |
| 2561 | 24130 | S | N856K | SNV | C | A | 2054 | 55.31 | Omicron BA.1 |
| 2561 | 24410 | S | N950N | SNV | G | A | 2073 | 42.35 | Delta AY* |
| 2561 | 24424 | S | Q954H | SNV | A | T | 2143 | 56.88 | All Omicron |
| 2561 | 24469 | S | N969K | SNV | T | A | 1809 | 55.67 | All Omicron |
| 2561 | 24503 | S | L981F | SNV | C | T | 1920 | 53.54 | Omicron BA.1 |
| 2561 | 25469 | ORF3a | S26L | SNV | C | T | 1680 | 44.76 | Delta AY* |
| 2561 | 25904 | ORF3a | S171L | SNV | C | T | 1064 | 41.17 | Both Delta and Omicron |
| 2561 | 26270 | E | T9I | SNV | C | T | 1115 | 58.21 | All Omicron |
| 2561 | 26530 | M | D3G | SNV | A | G | 357 | 50.14 | Omicron BA.1 |
| 2561 | 26577 | M | Q19E | SNV | C | G | 468 | 46.58 | All Omicron |
| 2561 | 26709 | M | A63T | SNV | G | A | 636 | 58.81 | All Omicron |
| 2561 | 26767 | M | I82T | SNV | T | C | 862 | 45.36 | Delta AY* |
| 2561 | 27638 | ORF7a | V82A | SNV | T | C | 1583 | 43.59 | Delta AY* |
| 2561 | 27752 | ORF7a | T120I | SNV | C | T | 1250 | 42.72 | Delta AY* |
| 2561 | 27987 | ORF8 | V32L | SNV | G | T | 1770 | 42.09 | Delta AY.57 |
| 2561 | 28248..28253 | ORF8 | D119- | Deletion | GATTTC | - | 1225 | 48.00 | Delta AY* |
| 2561 |  | ORF8 | F120- |  |  |  |  |  | Delta AY* |
| 2561 | 28311 | N | P13L | SNV | C | T | 1386 | 52.16 | All Omicron |
| 2561 | 28362..28370 | N | E31- | Deletion | GAGAACGCA | - | 1728 | 52.37 | All Omicron |
| 2561 |  | N | R32- |  |  |  |  |  | All Omicron |
| 2561 |  | N | S33- |  |  |  |  |  | All Omicron |
| 2561 | 28461 | N | D63G | SNV | A | G | 1365 | 44.62 | Delta AY* |
| 2561 | 28881 | N | R203M | SNV | G | T | 1708 | 46.43 | Delta |
| 2561 | 29402 | N | D377Y | SNV | G | T | 1379 | 43.22 | Delta |

*SNV: Single Nucleotide Variant; MNV: Multi Nucleotide Variant

**Table S2. List of detected mutations in two recombination cases**

| **Sample** | **Region** | **Gene** | **Acid amin change** | **Type** | **Reference** | **Allele** | **Coverage** | **Frequency** | **Variant** |
| --- | --- | --- | --- | --- | --- | --- | --- | --- | --- |
| 3220 | 670 | ORF1a | S135R | SNV | T | G | 240 | 100.00 | Omicron BA.2 |
| 3220 | 1820 | ORF1a | G519S | SNV | G | A | 187 | 93.05 | Both Delta and Omicron |
| 3220 | 2790 | ORF1a | T842I | SNV | C | T | 1057 | 99.72 | Omicron BA.2 |
| 3220 | 4184 | ORF1a | G1307S | SNV | G | A | 812 | 100.00 | Omicron BA.2 |
| 3220 | 4764 | ORF1a | H1500R | SNV | A | G | 752 | 99.20 | Delta |
| 3220 | 5184 | ORF1a | P1640L | SNV | C | T | 768 | 99.48 | Delta AY.57 |
| 3220 | 9867 | ORF1a | L3201P | SNV | T | C | 561 | 100.00 | NA |
| 3220 | 9891 | ORF1a | A3209V | SNV | C | T | 398 | 100.00 | Delta AY.57 |
| 3220 | 11418 | ORF1a | V3718A | SNV | T | C | 1250 | 99.84 | Delta AY.57 |
| 3220 | 11514 | ORF1a | T3750I | SNV | C | T | 1138 | 99.82 | Delta AY.57 |
| 3220 | 14408 | ORF1b | P314L | SNV | C | T | 837 | 100.00 | Both Delta and Omicron |
| 3220 | 15451 | ORF1b | G662S | SNV | G | A | 1248 | 99.84 | Delta AY* |
| 3220 | 16466 | ORF1b | P1000L | SNV | C | T | 1005 | 99.90 | Delta AY* |
| 3220 | 21614 | S | L18F | SNV | C | T | 439 | 99.77 | Delta AY* |
| 3220 | 21618 | S | T19R | SNV | C | G | 437 | 99.77 | Delta AY* |
| 3220 | 21846 | S | T95S | SNV | C | G | 535 | 100.00 | NA |
| 3220 | 21987 | S | G142D | SNV | G | A | 335 | 99.70 | Both Delta and Omicron |
| 3220 | 22029..22034 | S | E156- | Deletion | AGTTCA | - | 330 | 100 | Delta AY* |
| 3220 |  | S | F157- |  |  |  |  |  | Delta AY* |
| 3220 |  | S | R158G |  |  |  |  |  | Delta AY* |
| 3220 | 22200 | S | V213G | SNV | T | G | 317 | 100.00 | Omicron BA.2 |
| 3220 | 22578 | S | G339D | SNV | G | A | 509 | 100.00 | All Omicron |
| 3220 | 22674 | S | S371F | SNV | C | T | 572 | 99.65 | Omicron BA.2 |
| 3220 | 22679 | S | S373P | SNV | T | C | 556 | 100.00 | All Omicron |
| 3220 | 22686 | S | S375F | SNV | C | T | 558 | 99.46 | All Omicron |
| 3220 | 22688 | S | T376A | SNV | A | G | 558 | 100.00 | Omicron BA.2 |
| 3220 | 22775 | S | D405N | SNV | G | A | 628 | 72.93 | Omicron BA.2 |
| 3220 | 22786 | S | R408S | SNV | A | C | 574 | 100.00 | Omicron BA.2 |
| 3220 | 22812..22813 | S | K417T | MNV | AG | CT | 571 | 100.00 | Omicron BA.2.38 |
| 3220 | 22882 | S | N440K | SNV | T | G | 525 | 100.00 | Omicron BA.2 |
| 3220 | 22992 | S | S477N | SNV | G | A | 619 | 99.84 | All Omicron |
| 3220 | 22995 | S | T478K | SNV | C | A | 613 | 99.84 | Both Delta and Omicron |
| 3220 | 23013 | S | E484A | SNV | A | C | 616 | 99.68 | All Omicron |
| 3220 | 23040 | S | Q493R | SNV | A | G | 619 | 95.96 | Omicron BA.2 |
| 3220 | 23055 | S | Q498R | SNV | A | G | 622 | 100.00 | All Omicron |
| 3220 | 23063 | S | N501Y | SNV | A | T | 647 | 100.00 | All Omicron |
| 3220 | 23075 | S | Y505H | SNV | T | C | 617 | 99.35 | All Omicron |
| 3220 | 23403 | S | D614G | SNV | A | G | 1880 | 99.95 | Both Delta and Omicron |
| 3220 | 23525 | S | H655Y | SNV | C | T | 1842 | 100.00 | All Omicron |
| 3220 | 23599 | S | N679K | SNV | T | G | 1684 | 100.00 | All Omicron |
| 3220 | 23604 | S | P681H | SNV | C | A | 1642 | 99.94 | All Omicron |
| 3220 | 24424 | S | Q954H | SNV | A | T | 877 | 99.89 | All Omicron |
| 3220 | 24469 | S | N969K | SNV | T | A | 744 | 99.87 | All Omicron |
| 3220 | 25430..25435 | ORF3a | T14- | Deletion | TAACTT | - | 590 | 99.83 | NA |
| 3220 |  | ORF3a | L15- |  |  |  |  |  | NA |
| 3220 | 25469 | ORF3a | S26L | SNV | C | T | 573 | 100.00 | Delta AY* |
| 3220 | 25585 | ORF3a | L65V | SNV | C | G | 403 | 99.75 | Delta AY.57 |
| 3220 | 25919 | ORF3a | T176I | SNV | C | T | 571 | 99.82 | Delta AY.57 |
| 3220 | 26767 | M | I82T | SNV | T | C | 379 | 99.74 | Delta AY* |
| 3220 | 27382..27384 | ORF6 | D61L | MNV | GAT | CTC | 268 | 100.00 | Omicron BA.2 |
| 3220 | 28311 | N | P13L | SNV | C | T | 509 | 99.21 | All Omicron |
| 3220 | 28362..28370 | N | E31- | Deletion | GAGAACGCA | - | 471 | 99.79 | All Omicron |
| 3220 |  | N | R32- |  |  |  |  |  | Omicron BA.2 |
| 3220 |  | N | S33- |  |  |  |  |  | Omicron BA.2 |
| 3220 | 28881..28883 | N | R203K | MNV | GGG | AAC | 372 | 99.73 | All Omicron |
| 3220 |  | N | G204R |  |  |  |  |  |  |
| 3220 | 29510 | N | S413R | SNV | A | C | 555 | 100.00 | Omicron BA.2 |
| 3461 | 670 | ORF1a | S135R | SNV | T | G | 446 | 99.55 | Omicron BA.2 |
| 3461 | 1820 | ORF1a | G519S | SNV | G | A | 361 | 99.72 | Both Delta and Omicron |
| 3461 | 2790 | ORF1a | T842I | SNV | C | T | 2260 | 99.91 | Omicron BA.2 |
| 3461 | 4184 | ORF1a | G1307S | SNV | G | A | 1693 | 99.94 | Omicron BA.2 |
| 3461 | 4764 | ORF1a | H1500R | SNV | A | G | 2081 | 99.90 | Delta |
| 3461 | 5184 | ORF1a | P1640L | SNV | C | T | 1737 | 99.60 | Delta AY.57 |
| 3461 | 9867 | ORF1a | L3201P | SNV | T | C | 1339 | 99.93 | NA |
| 3461 | 9891 | ORF1a | A3209V | SNV | C | T | 1025 | 98.54 | Delta AY.57 |
| 3461 | 11418 | ORF1a | V3718A | SNV | T | C | 2507 | 99.76 | Delta AY.57 |
| 3461 | 11514 | ORF1a | T3750I | SNV | C | T | 2442 | 99.71 | Delta AY.57 |
| 3461 | 14408 | ORF1b | P314L | SNV | C | T | 2367 | 99.79 | Both Delta and Omicron |
| 3461 | 15451 | ORF1b | G662S | SNV | G | A | 2326 | 99.74 | Delta AY* |
| 3461 | 16466 | ORF1b | P1000L | SNV | C | T | 2065 | 99.85 | Delta AY* |
| 3461 | 17459 | ORF1b | P1331L | SNV | C | T | 1203 | 50.54 | NA |
| 3461 | 21614 | S | L18F | SNV | C | T | 1045 | 99.71 | Delta AY* |
| 3461 | 21618 | S | T19R | SNV | C | G | 1025 | 99.71 | Delta AY* |
| 3461 | 21846 | S | T95S | SNV | C | G | 1278 | 99.92 | NA |
| 3461 | 21987 | S | G142D | SNV | G | A | 820 | 99.76 | Both Delta and Omicron |
| 3461 | 22029..22034 | S | E156- | Deletion | AGTTCA | - | 830 | 99.88 | Delta AY* |
| 3461 |  | S | F157- |  |  |  |  |  | Delta AY* |
| 3461 |  | S | R158G |  |  |  |  |  | Delta AY* |
| 3461 | 22200 | S | V213G | SNV | T | G | 834 | 100.00 | Omicron BA.2 |
| 3461 | 22578 | S | G339D | SNV | G | A | 1146 | 99.83 | Omicron |
| 3461 | 22674 | S | S371F | SNV | C | T | 1129 | 99.20 | Omicron BA.2 |
| 3461 | 22679 | S | S373P | SNV | T | C | 1061 | 100.00 | All Omicron |
| 3461 | 22686 | S | S375F | SNV | C | T | 1056 | 100.00 | All Omicron |
| 3461 | 22688 | S | T376A | SNV | A | G | 1056 | 99.91 | Omicron BA.2 |
| 3461 | 22775 | S | D405N | SNV | G | A | 1144 | 99.83 | Omicron BA.2 |
| 3461 | 22786 | S | R408S | SNV | A | C | 1121 | 99.91 | Omicron BA.2 |
| 3461 | 22812..22813 | S | K417T | MNV | AG | CT | 1133 | 99.65 | Omicron BA.2.38 |
| 3461 | 22882 | S | N440K | SNV | T | G | 1048 | 99.62 | Omicron BA.2 |
| 3461 | 22992 | S | S477N | SNV | G | A | 1359 | 99.93 | All Omicron |
| 3461 | 22995 | S | T478K | SNV | C | A | 1332 | 99.92 | Both Delta and Omicron |
| 3461 | 23013 | S | E484A | SNV | A | C | 1288 | 99.92 | All Omicron |
| 3461 | 23040 | S | Q493R | SNV | A | G | 1277 | 99.53 | Omicron BA.2 |
| 3461 | 23055 | S | Q498R | SNV | A | G | 1275 | 99.92 | All Omicron |
| 3461 | 23063 | S | N501Y | SNV | A | T | 1354 | 100.00 | All Omicron |
| 3461 | 23075 | S | Y505H | SNV | T | C | 1269 | 99.84 | All Omicron |
| 3461 | 23403 | S | D614G | SNV | A | G | 3862 | 99.87 | Both Delta and Omicron |
| 3461 | 23525 | S | H655Y | SNV | C | T | 4006 | 99.95 | All Omicron |
| 3461 | 23599 | S | N679K | SNV | T | G | 3513 | 99.60 | All Omicron |
| 3461 | 23604 | S | P681H | SNV | C | A | 3427 | 99.97 | All Omicron |
| 3461 | 24424 | S | Q954H | SNV | A | T | 1875 | 99.84 | All Omicron |
| 3461 | 24469 | S | N969K | SNV | T | A | 1693 | 99.59 | All Omicron |
| 3461 | 25430..25435 | ORF3a | T14- | Deletion | TAACTT | - | 1284 | 99.45 | NA |
| 3461 |  | ORF3a | L15- |  |  |  |  |  | NA |
| 3461 | 25469 | ORF3a | S26L | SNV | C | T | 1310 | 99.69 | Delta AY* |
| 3461 | 25585 | ORF3a | L65V | SNV | C | G | 807 | 99.88 | Delta AY.57 |
| 3461 | 25919 | ORF3a | T176I | SNV | C | T | 1191 | 99.58 | Delta AY.57 |
| 3461 | 26767 | M | I82T | SNV | T | C | 792 | 99.87 | Delta AY* |
| 3461 | 27382..27384 | ORF6 | D61L | MNV | GAT | CTC | 619 | 100.00 | Omicron BA.2 |
| 3461 | 28311 | N | P13L | SNV | C | T | 1030 | 99.51 | All Omicron |
| 3461 | 28362..28370 | N | E31- | Deletion | GAGAACGCA | - | 1030 | 99.71 | All Omicron |
| 3461 |  | N | R32- |  |  |  |  |  | Omicron BA.2 |
| 3461 |  | N | S33- |  |  |  |  |  | Omicron BA.2 |
| 3461 | 28881..28883 | N | R203K | MNV | GGG | AAC | 725 | 99.72 | All Omicron |
| 3461 |  | N | G204R |  |  |  |  |  | All Omicron |
| 3461 | 29510 | N | S413R | SNV | A | C | 1090 | 99.72 | Omicron BA.2 |

**Supplementary figure**

**Figure S1. The phylogenetic tree on Nextclade database of two recombinant sequences with their modified sequences.** The colorful dots represent for the sequence. The original sequences: navi blue; the sequences with Delta regions only: green; the sequences with Omicron regions only: yellow.

**Figure S2. Mapped paired reads in the first breakpoint region.** The graph shows the distribution of mapped paired reads in the first breakpoint region which was extracted from CLC genomics workbench.
