# Supplementary figures and images for: Detection of co-infection and recombination cases with Omicron and local Delta variants of SARS-CoV-2 in Vietnam
Source: Sci Rep. 2024 Jun 20;14:14225. doi: 10.1038/s41598-024-64898-5 (PMC11190198; doi:10.1038/s41598-024-64898-5)

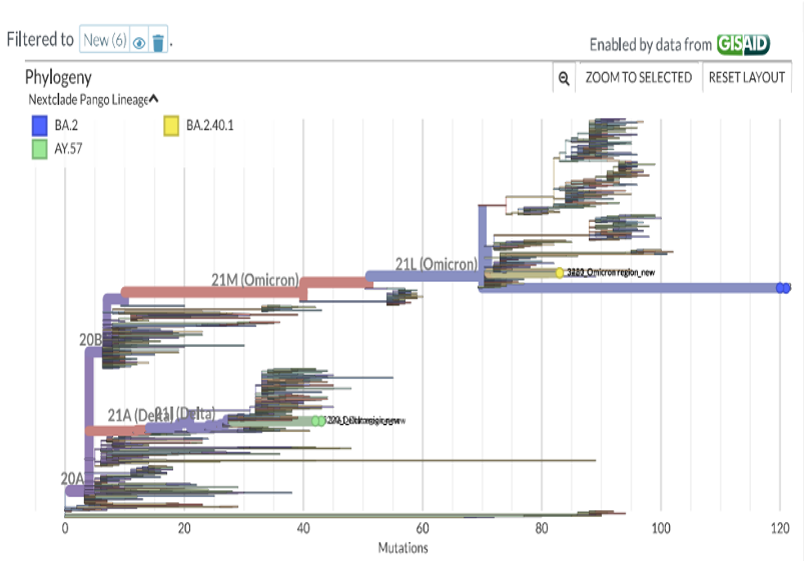

Supplement: Supplementary file 2 — Supplementary Information 2. [file 41598_2024_64898_MOESM2_ESM.tiff]

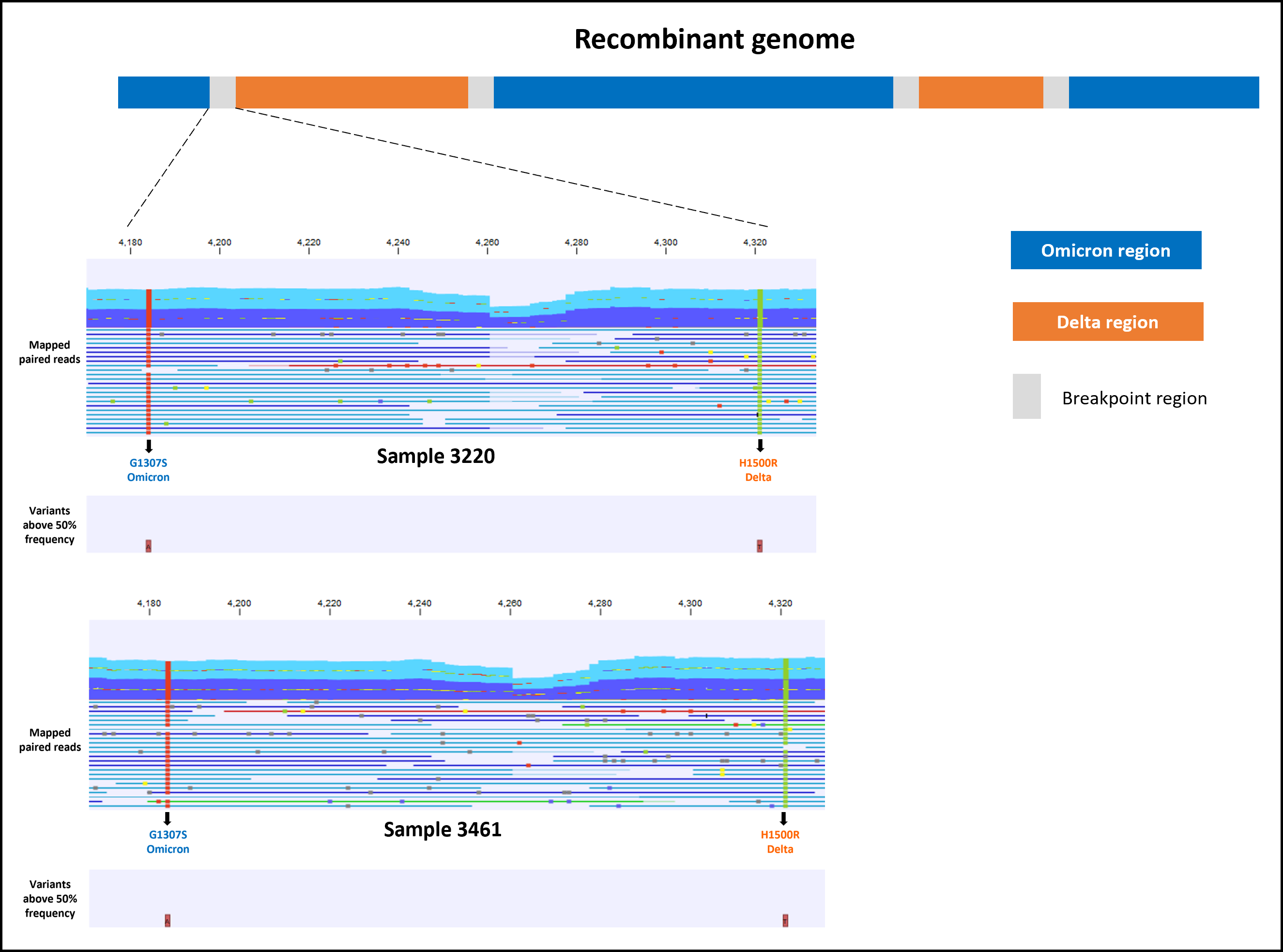

Supplement: Supplementary file 3 — Supplementary Information 3. [file 41598_2024_64898_MOESM3_ESM.tiff]
